# Modeling Partial Monosomy for Human Chromosome 21q11.2-q21.1 Reveals Haploinsufficient Genes Influencing Behavior and Fat Deposition

**DOI:** 10.1371/journal.pone.0029681

**Published:** 2012-01-20

**Authors:** Anna M. Migdalska, Louise van der Weyden, Ozama Ismail, Jacqueline K. White, Sanger Mouse Genetics Project, Gabriela Sánchez-Andrade, Darren W. Logan, Mark J. Arends, David J. Adams

**Affiliations:** 1 Wellcome Trust Sanger Institute, Hinxton, Cambridge, United Kingdom; 2 Department of Pathology, Addenbrooke's Hospital, University of Cambridge, Cambridge, United Kingdom; Institut Jacques Monod, France

## Abstract

Haploinsufficiency of part of human chromosome 21 results in a rare condition known as Monosomy 21. This disease displays a variety of clinical phenotypes, including intellectual disability, craniofacial dysmorphology, skeletal and cardiac abnormalities, and respiratory complications. To search for dosage-sensitive genes involved in this disorder, we used chromosome engineering to generate a mouse model carrying a deletion of the *Lipi*–*Usp25* interval, syntenic with 21q11.2-q21.1 in humans. Haploinsufficiency for the 6 genes in this interval resulted in no gross morphological defects and behavioral analysis performed using an open field test, a test of anxiety, and tests for social interaction were normal in monosomic mice. Monosomic mice did, however, display impaired memory retention compared to control animals. Moreover, when fed a high-fat diet (HFD) monosomic mice exhibited a significant increase in fat mass/fat percentage estimate compared with controls, severe fatty changes in their livers, and thickened subcutaneous fat. Thus, genes within the *Lipi*–*Usp25* interval may participate in memory retention and in the regulation of fat deposition.

## Introduction

The triplication of human chromosome 21, or subsets of genes mapped to the long arm of this chromosome, is known as Trisomy 21 or Down syndrome. In contrast, haploinsufficiency of genes on human chromosome 21 results in Monosomy 21. Complete Monosomy 21 typically results in prenatal death [1], [2], thus most cases that have been described are of partial or mosaic monosomy. Clinical phenotypes observed in patients with partial monosomies of chromosome 21 are very heterogeneous. Some patients show only mild to moderate intellectual disability, and have no other apparent dysmorphic or congenital malformations [3], [4], while others are diagnosed with a variety of severe clinical symptoms, such as profound intellectual disability, microcephaly, epilepsy, craniofacial, skeletal, cardiac and/or renal abnormalities and/or respiratory difficulties [5], [6], [7], [8], [9].

To date, four comprehensive studies have been performed using array comparative genomic hybridization (aCGH) and high-density single nucleotide polymorphism (SNP) genotyping to define the breakpoint regions present in patients with Monosomy 21, and to correlate these breakpoints with phenotype [7], [8], [9], [10]. However, it is clear from these studies that there is no direct correlation between the severity of the phenotype and the genes deleted; at least for patients carrying deletions proximal to the centromere (from the centromere to ∼31.2 Mb) and in the “medial” region of the chromosome (31.2–36 Mb). Patients carrying deletions proximal to the telomere (from ∼36−37.5 Mb to the telomere) typically show less severe clinical features (including mild to moderate intellectual disability, and either the complete absence of or only minor craniofacial abnormalities). Thus further investigation is required to identify the genes that are responsible for the clinical phenotypes observed in Monosomy 21 patients.

In parallel with the analysis of the genomes of patients with Monosomy 21 several mouse models of this condition have been developed [11], [12], [13], [14]. Synteny exists between human chromosome 21 (HSA21) and mouse chromosomes 16 (MMU16), 17 (MMU17) and 10 (MMU10). Specifically, about 23.2 Mb of human chromosome 21, from 21q11.2 to 21q22.3, is homologous to C3.1-C4 on MMU16, 1.1 Mb of 21q22.3 is homologous to the B1 band on MMU17 and 2.3 Mb of 21q22.3 region is syntenic to C1 on MMU10. A mouse model carrying a heterozygous deletion of MMU16 syntenic to the human region 21q22.12-q22.3 displayed reduced overall brain and hippocampus volume, but increased cerebellum volume. Nevertheless, these changes did not show any correlation with abnormal functioning of the hippocampus measured in the Morris water maze assay or by electrophysiology [13]. A mouse carrying a heterozygous 0.5 Mb deletion of MMU10, syntenic to the distal part of human region 21q22.3 located between the *PRMT2* and *COL6A1* genes, showed no gross morphological or behavioral anomalies, but exhibited an increased inflammatory reaction after intranasal lipopolysaccharide (LPS) administration [12]. In contrast, a mouse carrying a heterozygous 2.3 Mb deletion of MMU10, syntenic to the human region located between the *PRMT2* and *PDXK* genes, showed similarities to Monosomy 21-associated intellectual disability (with impairments in learning and memory), as did a mouse carrying a heterozygous deletion of a 1.1 Mb segment on MMU17, syntenic to the human region located between the *ABCG1* and *RRP1B* genes [14]. Interestingly, 13 out of 41 syntenic genes located on mouse chromosome 10, namely the genes located between the *Prmt2* and *Col6a1*, might be excluded as potential candidates for partial Monosomy 21-associated intellectual disability as deletion of these genes did not result in learning impairment in mice [12].

To our knowledge all currently available Monosomy 21 mouse models provide phenotypic data only on deletions syntenic to human regions located between 21q21.3 and the telomere [11], [12], [13], [14]. Thus, the contribution of additional regions/genes to Monosomy 21 remains unclear. To study this, we used chromosome engineering to generate a new mouse model of Monosomy 21, *Ms(Lipi-Usp25)1Dja* (abbreviated as *Ms1Dja*), carrying a deletion syntenic to 21q11.2-q21.1 in human, to assess the contribution of the 6 genes in this 1.6 Mb interval to the development of clinical features found in patients with Monosomy 21.

## Results

### Generation of monosomic mice for the 1.6 Mb *Lipi-Usp25* region

**Figure 1:**
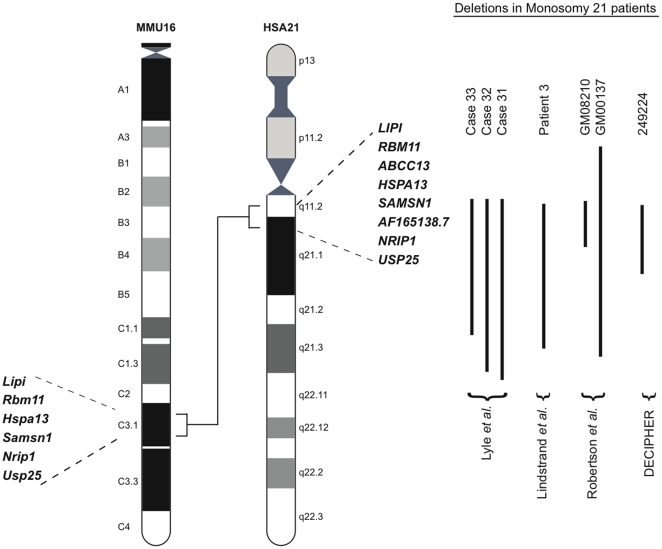
Schematic representation of HSA21 and the syntenic region on MMU16. The endpoints of the syntenic regions are indicated (*Lipi* and *Usp25*). Genes that map to the human 21q11.2-q21.1 region (NCBI build h36) and the C3.1 band on MMU16 (NCBI build m37) are listed. Studies describing partial Monosomy 21 patients with deletions involving the 21q11.2-q21.1 region (as indicated by the length of the black line) are shown.

**Figure 2:**
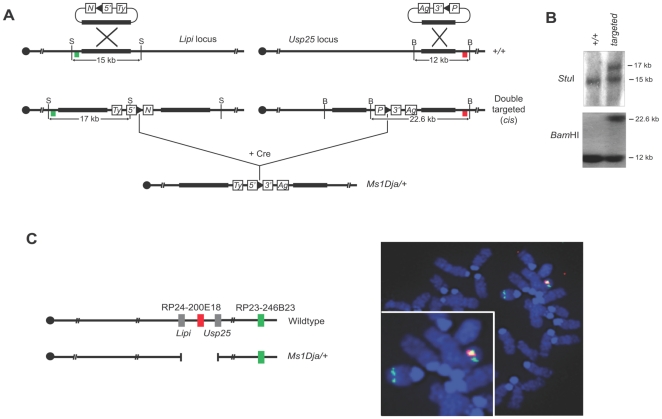
Generating a 1.6 Mb deletion between the *Lipi* and *Usp25* loci. (A) The targeting vectors contain a *loxP* site (arrowhead), a selectable antibiotic resistance gene (*N* (neomycin) or *P* (puromycin)), a coat color marker (*Ty* or *Ag*) and part of the *Hprt* gene (5′ or 3′) were targeted as shown. The colored boxes (green and red) indicate the location of the probes (5′ and 3′, respectively) used for Southern blotting (B) The targeting events were confirmed by Southern blot analysis showing an additional *Stu*I fragment of 17 kb compared with the wildtype allele (15 kb) for the *Lipi* locus and an additional *BamH*I fragment of 22.6 kb compared with the wildtype allele (12 kb) for the *Usp25* locus. B, *BamH*I; S, *Stu*I; P, puromycin; N, neomycin; Ty, *Tyrosinase*; Ag, *Agouti*. (C) Interphase FISH analysis with BAC probes that map in the region of the deletion (red) and outside (green). Chromosomes from the ES cells double-targeted in *cis* (*Ms1Dja/+*) showed two green and only red signal due to the deletion of the *Lipi–Usp25* region, while chromosomes from the wildtype ES cells showed two green and two red signals.

The *Lipi* and *Usp25* genes are located at the proximal and distal ends of a 1.6 Mb region in the C3.1 band of MMU16, which is syntenic to 21q11.2-q21.1 in humans (**Figure 1**). This region on HSA21 contains 8 genes (NCBI build h36), whereas the syntenic region in MMU16 contains only 6 genes (NCBI build m37) orthologous to their HSA21 counterparts as there are no annotated murine orthologs of *ABCC13* and *AF165138.1*. To generate the deletion we used chromosomal engineering [15]. Briefly, E14TG2a ES cells were sequentially electroporated with targeting vectors containing a portion of the *Hprt* selection cassette (5′ or 3′ *Hprt*), a *loxP* site and a coat color marker (Agouti or Tyrosinase). The targeting vector containing the 5′ *Hprt* cassette (MICER clone: MHPN69h23 [16] was inserted proximal to *Lipi* and the targeting vector containing the 3′ *Hprt* cassette (pUSP-3HPAg) was inserted distal to *Usp25* (**Figure 2A**). The correct insertion of both targeting vectors was confirmed by Southern blot analysis on *Stu*I- or *Bam*H1-digested gDNA from ES clones using a 5′ and 3′ Southern external probe, respectively (**Figure 2B**). ES cell clones that were targeted on the same chromosome (in *cis*) were electroporated with a Cre-expression vector, and selected in medium with hypoxanthine, aminopterin and thymidine (HAT) to isolate ES clones carrying a chromosomal deletion generated *via loxP* sites recombination (**Figure 2A**). The deletion allele was designated *Ms(Lipi-Usp25)1Dja* (abbreviated as *Ms1Dja*). The presence of the deletion in *Hprt*-resistant ES clones was confirmed by fluorescence *in situ* hybridization (FISH) (**Figure 2C**). The positive ES clones were injected into C57BL/6-*Tyr^c-Brd^* blastocysts to generate chimeras, which transmitted the *Ms1Dja* allele to their progeny. After the germline transmission, monosomic mice were backcrossed to and maintained on the C57BL/6-*Tyr^c-Brd^* background.

#### Homozygous deletion of the Lipi–Usp25 region results in embryonic lethality

**Figure 3:**
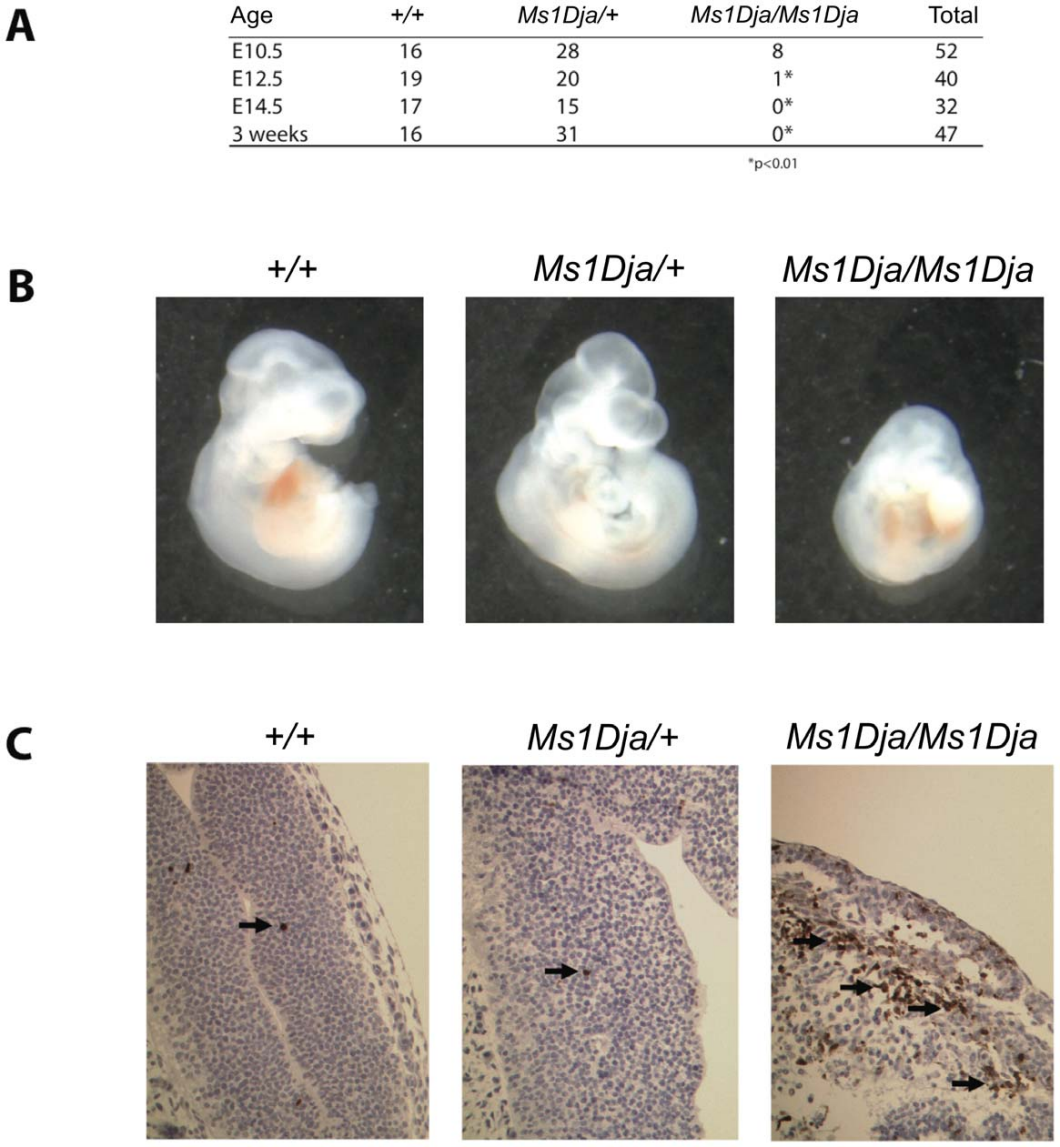
Embryonic lethality of mice homozygous for the *Lipi-Usp25* deletion. (**A**) Genotyping of E10.5, E12.5, E14.5 embryos, and 3-week old mice from monosomic *Ms1Dja* intercrosses. Asterisk indicates *P*<0.01 (Chi-square test). (**B**) Photos of wildtype (*+/+*), monosomic (*Ms1Dja/+*) and nullisomic (*Ms1Dja/Ms1Dja*) embryos at E10.5. Longitudinal measurements are 4 mm, 4 mm and 2.7 mm for the wildtype, monosomic and nullisomic embryos, respectively. (**C**) Immunohistochemical analysis using a Cleaved Caspase-3 (Asp175) antibody showed markedly increased number of apoptotic cells (brown staining; highlighted by black arrows) in the neural tube of nullisomic (*Ms1Dja/Ms1Dja*) E10.5 embryos. Images are representative and taken at ×200 magnification.

Monosomic (heterozygous) *Ms1Dja* mice were viable and fertile and did not show any overt phenotypic abnormalities. In intercrosses of monosomic *Ms1Dja* mice, no nullisomic (homozygous) *Ms1Dja* mice were recovered at birth out of 47 animals generated (**Figure 3A**). To determine the age of embryonic lethality of the nullisomic mice, embryos from monosomic intercrosses were collected at different timepoints during gestation (E10.5, E12.5 and E14.5). Nullisomic embryos were identified at E10.5 at low but still within normal Mendelian ratios (8/52 embryos), whereas only one very necrotic one was found at E12.5 (1/40 embryos, *P*<0.01), although increased numbers of reabsorbed embryos were observed, and none were recovered at E14.5 (0/32 embryos, *P*<0.01); **Figure 3A**. Even at E10.5, the nullisomic embryos were markedly smaller than their littermates (**Figure 3B**) and showed an increased number of apoptotic cells in the neural tube (as determined by hematoxylin and eosin-stained sections and Cleaved Caspase-3 immunohistochemistry; **Figure 3C**) with prominent degeneration of the neural tube as well as some other embryonic structures. This indicates that the majority of embryonic lethality in the nullisomic embryos happened on or before day E10.5 and was probably due to a defect in neural tube formation.

#### Phenotyping and dysmophology analysis of monosomic Ms1Dja mice

To determine whether clinical features of patients with Monosomy 21 could be observed in the monosomic *Ms1Dja* mice, 14 monosomic mice and 14 of their wildtype littermates fed a high-fat diet (HFD) from the age of 4 weeks were subjected to a series of tests designed to analyze their morphology, motor skills, behavior, neuromuscular function, pain perception, hearing, metabolism and hematology. **Table S1** provides a short summary of the phenotypic tests performed. The monosomic mice were viable, fertile and did not show any overt phenotype upon observation. They showed no gross motor or neurological abnormalities and had a similar level of pain perception, muscle strength and hearing compared with control animals (wildtype littermates), showing that the monosomic mice had no gross behavioral or neuromuscular defects.

The general dysmorphology and eye morphology tests only found a difference between the controls and monosomic mice with respect to eye pigmentation. Pigmentation of the eyes and abnormal iris pigmentation was observed in albino monosomic mice. However, this can be explained by the presence of coat colour markers (tyrosinase and agouti) present in the constructs used for gene-targeting in the ES cells. Thus, it was concluded that the monosomic mice showed no gross dysmorphology.

### Behavioral phenotyping of monosomic *Ms1Dja* mice

**Figure 4:**
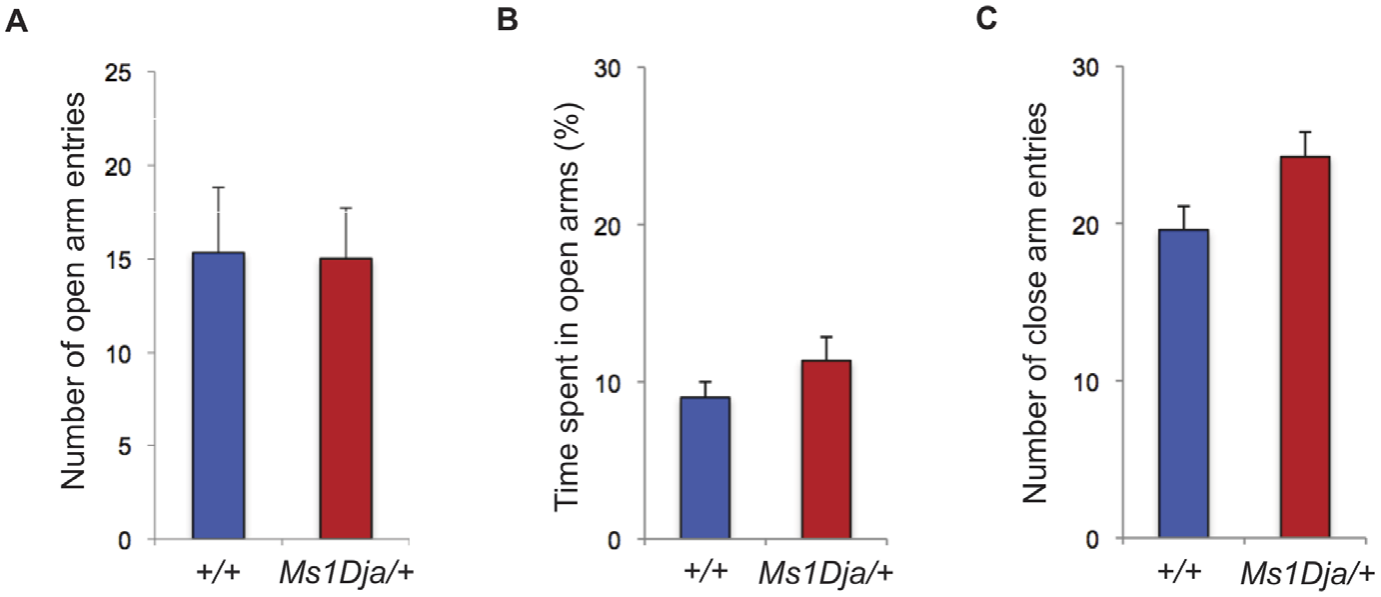
Elevated plus maze test. (**A**) Percentage of time spent in the open arms. (**B**) Number of entries into the open arms. (**C**) Number of entries into the closed arms. Both monosomic (*Ms1Dja/+*, n = 12) and control (*+/+*, n = 12) mice spent similar amount of time in the open arms and had comparable number of entries into the open and closed arms. Tested with two-tailed Student's *t*-test. The error bars represent the standard error of the mean.

**Figure 5:**
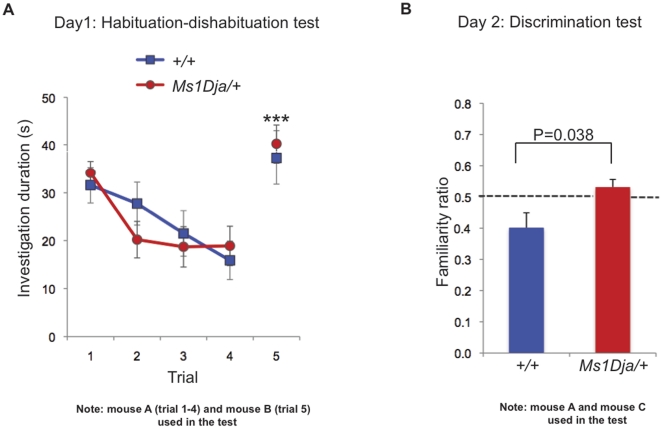
Social recognition test. (**A**) Habituation-dishabituation test. Both monosomic (*Ms1Dja/+*, n = 10) and control (*+/+*, n = 8) mice recognized two different stimulus animals (mouse A and mouse B), as shown by decline in the investigation time over trials 1 to 4 when they were repetitively presented the same (familiar) stimulus animal (mouse A) and increase in the investigation time on trial 5 when they were presented with a novel stimulus animal (mouse B) (trial 4 *vs* trial 5, *** *P*<0.0001, *post-hoc* analysis after two-way anova, the error bars represent the standard error of the mean). (**B**) Discrimination test. 24 hours after the habituation-dishabituation test. When given a choice between the familiar stimulus mouse (mouse A, thus the mouse used for trials 1 to 4 on day 1) and a new unfamiliar mouse (mouse C), control mice spent significantly less amount of time investigating the familiar stimulus mouse (mouse A) than the unfamiliar one (mouse C). Monosomic (*Ms1Dja/+*) did not recognize the familiar from the unfamiliar animal, shown by a familiarity ratio close to 0.5 (“chance”, dotted line). Control animals had a significantly smaller familiarity ratio than monosomic (*Ms1Dja/+*) mice (two-tailed Student's *t*-test, the error bars represent the standard error of the mean). Five animals (two mutants and three wildtypes) were taken out of the analysis because of their low investigation times (less than 10 seconds on trial 1).

To further explore behavioral and cognitive function, 12 monosomic mice and 12 wildtype littermate controls were subjected to an elevated plus maze assay to test for anxiety, and a social recognition paradigm with a long-term social memory component. Both monosomic and wildtype animals showed a preference for the closed arm compared to the open arm of the elevated plus maze, as measured by the amount of time spent in the open arm, and the number of entries into the open and closed arm (**Figure 4A, 4B, 4C**; two-way ANOVA: repeated measures for *Trial*, F_4,64_ = 25.86, *P*<0.0001, effect for *genotype* F_1,64_ = 11.26, *P* = 0.943). The social recognition test showed that the monosomic animals were able to recognize two different (sedated) male stimulus animals (mouse A and mouse B) (**Figure 5A**; two-way ANOVA: repeated measures for *Trial*, F_4,64_ = 25.86, *P*<0.0001, trial 4 vs trial 5, *P*<0.05, *post hoc* analysis). Both groups of mice had similar initial levels of investigation, and spent increasingly lower amounts of time investigating the repeatedly presented stimulus animal (mouse A) (**Figure 5A**; two-way ANOVA effect for *genotype* F_1,64_ = 11.26, *P* = 0.943, interaction *Trial*×g*enotype* F_4,64_ = 1.762, *P* = 0.1475). Both of these results suggest normal levels of anxiety and social interaction. However, when subjected to a 24- hour social memory retention test, monosomic mice were less capable of distinguishing a familiar stimulus animal (mouse A) from an unfamiliar one (mouse C) (**Figure 5C**; ratio of investigation het vs wt, *P*<0.05 compared with two-tailed Student's *t*-test). Wildtype animals, in contrast, retained the memory of the familiar stimulus animal (mouse A) and investigated it less frequently. To ensure this deficit was cognitive, and not simply a consequence of diminished olfactory capacity in monosomic *Ms1Dja* mice, we ascertained whether they could recognize odors in the absence of other sensory cues. Indeed, monosomic and wildtype animals were equally capable of detecting odors and of discriminating between them (**Figure S1**).

### Analysis of the body composition of monosomic *Ms1Dja* mice

**Figure 6:**
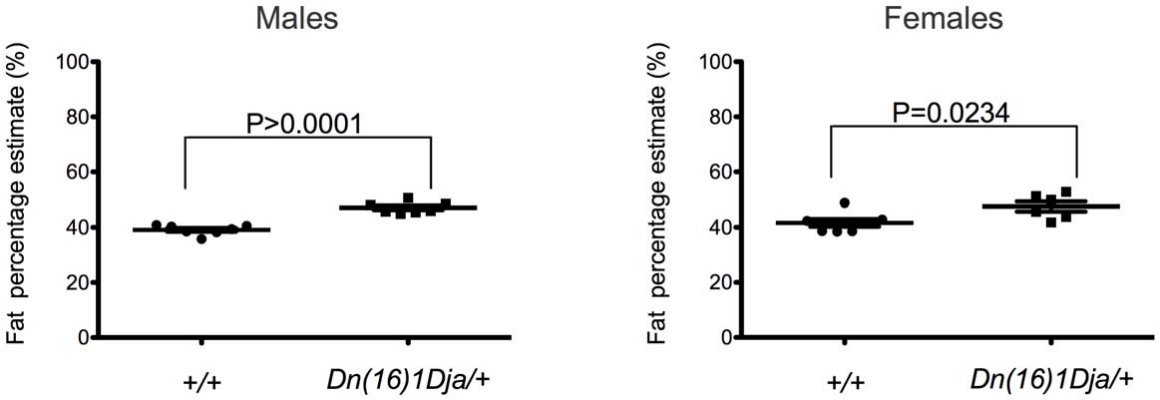
Fat percentage estimate of 14-week old control and monosomic mice fed a high-fat diet. DEXA results showing fat percentage estimate in 14-week old male (n = 7) and female (n = 7) control (*+/+*) and male (n = 7) and female (n = 6) monosomic (*Ms1Dja/+*) littermates fed a high-fat diet. Asterisks indicate statistical significance (two-tailed Student's *t*-test). The error bars represent the standard deviation of the measurements.

Analysis of the body fat composition and bone mineral density of the mice by dual energy X-ray absorptiometry (DEXA) revealed that monosomic mice exhibited a statistically significant increase in fat mass and fat percentage estimate compared with controls (**Figure 6**). It is important to note that this was not due to any significant differences in the weight of the monosomic mice from the controls (data not shown). However, despite the increased fat deposition in the monosomic mice, indirect calorimetry and glucose tolerance tests, as well as the analysis of whole blood and plasma clinical chemistry parameters, including the analysis of the triglycerides, cholesterol, non-esterified free fatty acids, and low and high density lipoproteins, revealed no significant differences between the monosomic mice and controls (data not shown). There was also no significant difference in food intake between the two groups.

Thus taken together, we conclude that whereas monosomy of the *Lipi–Usp25* region does not have a dramatic impact on morphology, motor skills, behavior, whole-body metabolism and hematological parameters, it does appear to affect memory retention and the deposition and/or metabolism of fat.

#### Increased body fat percentage in monosomic mice fed on a high-fat diet

**Figure 7:**
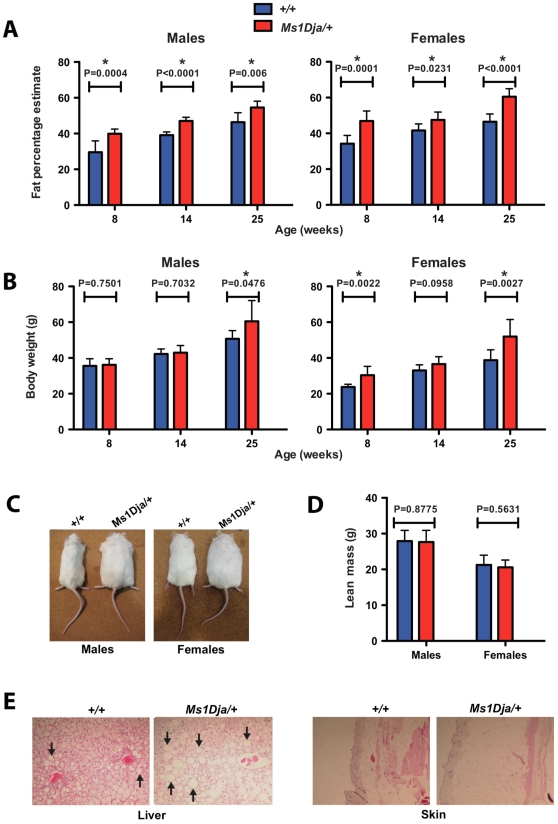
Analysis of high-fat diet-fed monosomic mice at different ages. DEXA results showing (**A**) fat percentage estimate and (**B**) body weight measurements in 8-, 14- and 25-week old male (n = 8, n = 7 and n = 8, respectively) and female (n = 8, n = 7 and n = 10, respectively) control (*+/+*) and male (n = 9, n = 7 and n = 7, respectively) and female (n = 10, n = 6 and n = 9, respectively) monosomic (*Ms1Dja/+*) littermates fed a high-fat diet. Asterisks indicate statistical significance (two-tailed Student's *t*-test). The error bars represent the standard deviation of the measurements. (**C**) Photos of 25-week old male and female control (+/+) and monosomic (*Ms1Dja/+*) littermates fed a high-fat diet. (**D**) DEXA results showing lean mass measurements in 25-week old male (n = 8) and female (n = 10) control (*+/+*) and male (n = 7) and female (n = 9) monosomic (*Ms1Dja/+*) littermates fed a high-fat diet. Analyzed with two-tailed Student's *t*-test. The error bars represent the standard deviation of the measurements. (**E**) Haematoxylin and eosin-stained liver and skin sections from 25-week old wildtype (+/+) and monosomic (*Ms1Dja/+*) littermates fed a high-fat diet. Note markedly increased number and size of adipose cells in liver sections from monosomic (*Ms1Dja/+*) mice compared to wildtype (+/+) littermates (highlighted by black arrows). Images are representative and taken at ×100 magnification.

Following on from the preliminary DEXA results that suggested that monosomic mice fed on a high-fat diet (HFD) exhibit a statistically significant increase in fat mass/fat percentage estimate at 14 weeks, we analyzed monosomic and control mice fed on a HFD at additional time points, namely at 8 and 25 weeks. As expected both monosomic and control mice showed a gradual increase in fat mass/fat percentage estimate with age (**Figure 7A**). However, the monosomic mice showed a significantly higher increase in fat mass/fat percentage estimate compared with controls (at both 8 and 25 weeks of age).

We next compared the average weight of the two groups of mice at each time point to see if the increase in fat mass/fat percentage estimate was reflected in the overall body weight gain (**Figure 7B**). Whilst no significant differences were observed in the average weight of the monosomic mice compared with controls at 8 and 14 weeks of age, the 25-week old monosomic mice showed a significant increase in body weight over the controls (**Figures 7B and 7C**). The body weight gain observed in 25-week old monosomic mice is most likely due to their increased fat deposition as their lean mass was not significantly changed when compared to the controls (**Figure 7D**). This increased fat deposition resulted in fatty changes in the livers of the mice, with severe to very severe fatty changes observed in the monosomic mice compared to only mild to moderate fatty changes in controls (**Figure 7E**). Similarly, 25-week old monosomic mice fed on a HFD showed a markedly thicker layer of subcutaneous fat and enlarged adipose cells in the skin compared with controls (**Figure 7E**).

#### The increased fat deposition in monosomic mice is diet-induced

**Figure 8:**
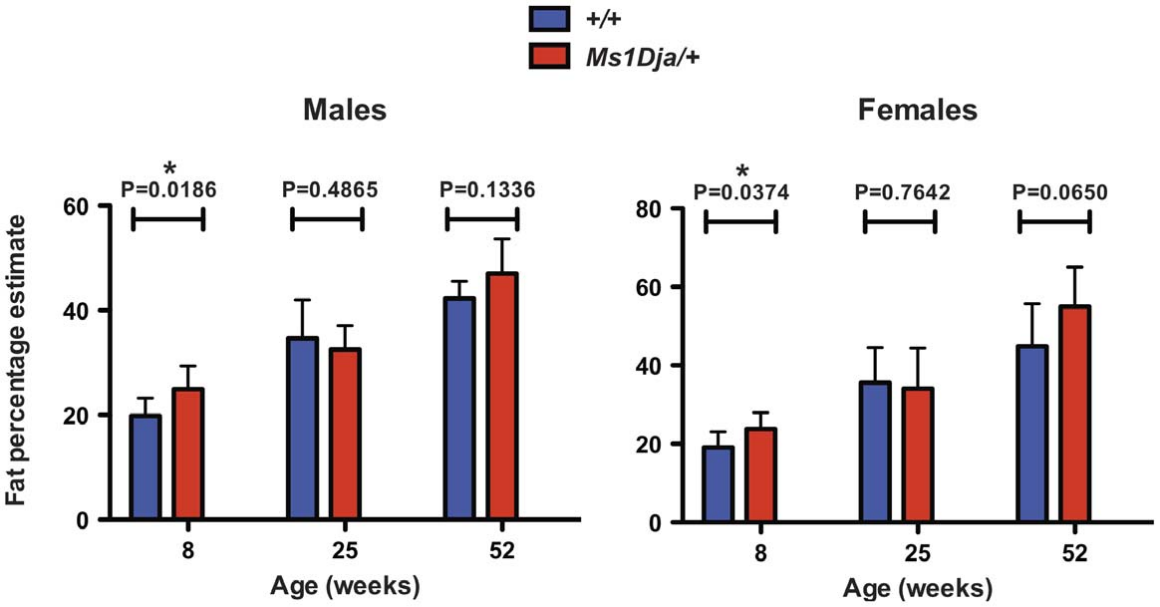
Fat percentage estimate of control and monosomic mice fed a normal-fat diet. DEXA results showing fat percentage estimate in 8-, 25- and 52-week old male (n = 8, n = 10 and n = 6, respectively) and female (n = 8, n = 10 and n = 8, respectively) control (*+/+*) and male (n = 9, n = 7 and n = 9, respectively) and female (n = 10, n = 9 and n = 9, respectively) monosomic (*Ms1Dja/+*) littermates fed a normal-fat diet. Asterisks indicate statistical significance (two-tailed Student's *t*-test).

In order to determine if the HFD was the causal factor for the increased fat deposition, monosomic and control mice were fed on a normal-fat diet (NFD) were subjected to DEXA analysis at 8 and 25 weeks (**Figure 8**). At 8 weeks of age, the monosomic mice showed a significant increase in fat percentage estimate compared to controls, although this increase was more obvious on a HFD. However, NFD monosomic mice at 25 weeks of age did not show a significantly increased fat mass/fat percentage estimate compared with the littermate controls. Concomitant with this, 1-year old monosomic also did not show a significant increase in fat mass/fat percentage estimate compared with controls. Similarly, histopathological analysis of the livers from NFD monosomic mice at 8-, 25-weeks and 1-year of age exhibited comparable levels of fatty changes with those observed in the controls, as no fatty changes were seen in livers of either monosomic or wildtype mice at either 8 or 25 weeks of age, and at 1 year the degree of hepatic fatty change ranged from mild through moderate to severe in both monosomic and wildtype mice (data not shown). Thus, it seems the HFD is the causal factor of the increased fat mass/fat percentage estimate in monosomic mice.

#### Expression analysis of the genes from the deleted Lipi−Usp25 interval in subcutaneous adipose tissue

We performed quantitative RT-PCR (qRT-PCR) to investigate whether any of the six genes from the deleted *Lipi−Usp25* interval showed a differential expression between adipocytes from monosomic and control mice fed on a HFD (n = 8 per genotype at 16 weeks of age). Of the genes analyzed by qRT-PCR, four were significantly down-regulated (*Hspa13*, *Samsn1*, *Nrip1* and *Usp25*; *P* = 0.0007, *P* = 0.0057, *P* = 0.0361 and *P* = 0.0379, respectively; **Figure S2**), and two were not expressed in adipose tissue (*Lipi* and *Rbm11*).

## Discussion

We report on the generation and phenotypic characterization of the monosomic *Ms1Dja* mouse, a model of Monosomy 21 covering the 1.6 Mb *Lipi*–*Usp25* region. The deleted interval is syntenic to the centromeric part of the HSA21, and contains 6 genes that are conserved between human and mouse. Homozygous deletion of this region (nullisomy) resulted in mid-gestational embryonic lethality mostly due to neural tube degeneration with evidence of prominent apoptosis, indicating that at least one of the genes in this region is required for development and maintenance of the neural tube. However, as none of the 6 genes in this region have been reported to be involved in neural tube development (and all are normally expressed in the developing brain of the mouse embryo at both E9.5 and E10.5 [17], it is hard to speculate which gene (or genes) might be responsible. However, given that homozygous null *Samsn1* and *Nrip1* mice are viable [18], [19], it is unlikely that loss of these genes causes the embryonic lethality observed.

Detailed phenotypic analysis of monosomic *Ms1Dja* mice, found them to be viable, fertile, and to display no obvious differences from wildtype littermate controls in terms of morphology, motor skills, gross behavior, and both clinical chemistry and hematological parameters were normal. They did, however, show deficits in long-term memory retention in a socially relevant testing paradigm. Social recognition is hippocampus-dependent [20] and has been used for learning and memory testing [21], [22] and for assessing cognitive impairment in mice [23]. Thus haploinsufficiency of the genes mapped within the deletion may contribute to the intellectual disability of humans with Monosomy 21, but they do not model the other clinical phenotypes commonly seen in Monosomy 21 patients. However, given all currently known Monosomy 21 patients with “centromeric” deletions carry rearrangements spanning much larger regions of HSA21 than represented in our model, we cannot rule out the possibility that genes in the *Lipi*-*Usp25* region work in synergy contribute to the cognitive phenotype observed in these patients. In addition, there are 2 genes in the 21q11.2-q21.1 region of HSA21 for which there are no mouse orthologs annotated, namely *ABCC13* and *AF165138.7*. Our model does not account for the possible roles that these genes may play in Monosomy 21.

Monosomic mice fed a HFD showed significantly increased fat percentage estimates at 8, 14, and 25 weeks of age compared with controls. However, these significantly increased fat percentage estimates over controls were not observed when the monosomic mice were fed a NFD (except at 8 weeks of age, which might be considered as an early phenotype variation). Considering the fact that the overall fat percentage estimate was much higher in both groups of mice on a HFD (containing 21% crude fat) compared to a NFD (containing 9% crude fat), the HFD can be regarded as an environmental factor that increases fat percentage estimates in the mice. Given that monosomic mice showed significantly higher percentage fat estimates than wildtype controls, it is clear that there is also a genetic factor affecting this phenotypic change. However, although the interaction between genetic susceptibility *loci* and environmental factors in the development of obesity have been shown in different analyses performed both in humans and mice [24], [25], [26], [27], [28], [29], [30], to our knowledge, this interaction has not been shown before in patients with deletions encompassing the 21q11.2-q21.1 region.

Although four published studies have reported on the presence or absence of obesity in Monosomy 21 patients with deletions encompassing the 21q11.2-q21.1 region [4], [31], [32], [33], only one found a positive correlation; two cases that presented with mild intellectual disability, some facial abnormalities, and obesity [33]. Unfortunately, without knowledge of the diet of these individuals, we can only speculate whether the observed obesity in these patients was diet induced.

There are 6 genes that are conserved between human and mouse in the *Lipi-Usp25* region: *Lipi*, *Rbm11*, *Hspa13*, *Samsn1*, *Nrip1* and *Usp25*. To investigate potential molecular mechanisms leading to HFD-induced obesity in monosomic *Ms1Dja* mice, we performed qRT-PCR analysis on all the genes from the deleted interval in adipocytes from monosomic and wildtype littermates. We found four of them to show a significant down-regulation in monosomic mice relative to controls (*Hspa13*, *Samsn1*, *Nrip1* and *Usp25*) and two of them were not expressed in subcutaneous adipose tissue (*Lipi* and *Rbm11*). However, as none of the four genes expressed in adipocytes have been reported to be involved in the regulation of adipocyte metabolism, it is hard to speculate if any of the down-regulated genes might be responsible for HFD-induced increase in fat deposition our monosomic mice.

To-date, homozygous null mice for *Samsn1* and *Nrip1* [18], [19] and a mouse model carrying a deletion of the exon 10 of the mutant *Lipi* (*Lpd*, *Lpdl*) locus [34] have been generated. *Samsn1* knockout mice were viable and did not show any overt phenotypic abnormalities, but displayed enhanced adaptive immunity [19]. However, body weight and resistance to HFD-induced obesity and hepatic steatosis have not been specifically analyzed in either heterozygous or homozygous *Samsn1* mice. Homozygous *Nrip1* (*Nrip1^−/−^*) mice fed a NFD showed a 20% reduction in body weight and remained leaner than controls even when fed a HFD, despite the fact that *Nrip1* affects the function of adipose tissue by blocking both mitochondrial respiration and energy uncoupling and prevents expression of key regulatory genes in white adipose tissue (WAT) [35]. In addition, *Nrip1^−/−^* mice were resistant to both age-induced hepatic steatosis and HFD-induced fatty changes in the liver (although this could be explained by the increased expression of genes involved in energy expenditure and mitochondrial uncoupling, and the decreased expression of genes encoding lipogenic enzymes) [35]. However, body weight and resistance to HFD-induced obesity and hepatic steatosis was only demonstrated in *Nrip1* ^−/−^ mice and *Nrip1^+/^* ^−^ animals have not been studied. Homozygous *Lipi* (*Lipi^lpd1^/Lipi^lpd1^*) mice fed on a NFD showed hepatic steatosis and hypertriglyceridemia, while heterozygous *Lipi* (*Lipi^lpd1^/+*) mice did not display any of such phenotypic abnormalities [34]. No data for body weight or resistance to HFD-induced obesity have been available for either *Lipi^lpd1^/Lipi^lpd1^* or *Lipi^lpd1^/+* mice. Interestingly, sequencing of the human *LIPI* (*LPDL*) gene in 186 individuals with hypertriglyceridemia and 232 controls identified a nonsynonymous coding SNP 164G>A (C55Y) in two patients withhypertriglyceridemia, indicating that this rare missense mutation in the exon 2 of the *LIPI* gene might be associated with an elevated triglycerides level [34]. To that end, we evaluated the level of triglicerides in monosomic *Ms1Dja* and wildtype littermates, but observed no significant differences between monosomic mice and controls. As none of the other 3 genes in the *Lipi-Usp25* region are known to be involved in lipid and carbohydrate metabolism or maintenance of energy homeostasis, it is difficult to hypothesize which gene (or genes) is responsible for HFD-induced increase in fat deposition and fatty changes in livers of our monosomic mice. Importantly, although the *Ms1Dja* mice were backcrossed to C57BL/6-*Tyr^c-Brd^* for several generations prior to phenotyping, they will retain some ES derived 129P2/OlaHsd DNA linked to the targeted locus that may carry with it variants that contribute to the phenotypes we observe here.

Monosomy 21 is a rare human disease with variable clinical appearances due to differing gene dosage errors on chromosome 21 resulting in phenotypes including intellectual disability, dysmorphology, and cardiac and/or renal abnormalities [5], [6], [7], [8], [9]. Attempts to generate genotype-phenotype correlations in this disease have been complicated by both the small number of patients available for study (there is a lack of informative sets of partial Monosomy 21 patients [7]) and the fact that some patients with Monosomy 21 also carry anomalies involving other human chromosomes, such as segmental trisomies and deletions [7], [36]. We show here that haploinsufficiency of a gene (or genes) in the *Lipi-Usp25* region of MMU16, syntenic to human 21q11.2-q21.1, results in a high-fat diet-induced increase in fat deposition and a deficit in memory retention in monosomic mice. Further studies will be required to understand the molecular causes of these phenotypes.

## Materials and Methods

### Ethics Statement

Experiments were approved by both the Home Office, and the Local Institutional Ethics Committee.

### Gene targeting in ES cells and generation of deletion mice

The *5′HPRT* MICER targeting vector MHPN69h23 [16] was linearized with *Nhe*I and electroporated into E14Tg2a ES cells (129P2/OlaHsd) which were cultured as described previously [37]. Stable integrants were selected in 175 µg/ml G418. Positive clones were identified by Southern blotting on *Stu*I-digested gDNA (using a probe amplified from E14Tg2a gDNA using primers: 5′-AGG CAA AAA CCA AGA CCT CA-3′ and 5′-ATG GTG GCA ATG TTC TCA CA-3′). The *3′HPRT* targeting vector (pUSP-3HPAg) was constructed using recombineering to capture a 6,963 bp fragment telomeric to *Usp25* from 129Sv BAC clone: bMQ134j07 [38]. The 3′ recombineered targeting vector was linearized with *Swa*I and electroporated into MHPN69h23-targeted ES cells, which were subsequently selected in 3 µg/ml puromycin. Southern blotting was performed on *Bam*HI-digested DNA (using a probe amplified from E14Tg2a gDNA using primers: 5′-GTG CCC ACA TGG TTT TCT TT-3′ and 5′-CAA CTC TCG CCT CAC ACA AA-3′). To determine if the *5′HPRT* and *3′HPRT* targeting vectors were targeted in *cis-* or in *trans-* we electroporated double-targeted ES cell clones with the pOG231 Cre-expression vector [39] and selected them in hypoxanthine, aminopterin, and thymidine (HAT) medium as described previously [40], [41]. Clones of ES cells that carried the desired deletion between *Lipi* and *Usp25* generated by *cis*-recombination were identified and confirmed by PCR using the primers: 5′-AAG GGT GTT TAT TCC CCA TGG ACT AAT TAT G-3′ and 5′-CCT TCA TCA CAT CTC GAG CAA GAC GTT CAG-3′ (presence of a 1.7 kb band confirmed the *cis* orientation). The deletion allele was designated *Ms(Lipi-Usp25)1Dja* (abbreviated as *Ms1Dja*). ES cell clones carrying *Ms1Dja* were injected into C57BL/6-*Tyr^c-Brd^* blastocysts and transmitted through the germline. After the germline transmission of the deletion allele from the chimeric founder mice, the monosomic mice were backcrossed to and maintained on a C57BL/6-*Tyr^c-Brd^* background. All mice were housed and procedures carried out in accordance with Home Office guidelines (United Kingdom). Experiments were approved by both the Home Office, and the Local Institutional Ethics Committee.

### Fluorescent in situ hybridization (FISH)

Chromosome spreads of activated splenocytes were performed as described previously [42]. Mouse BAC clones were chosen to be located inside (RPCI24-200E18) or outside (RPCI24-246B23) the deletion region (http://bacpac.chori.org/mmouse24.htm). One microgram of mouse BAC DNA was used to generate DNA probes labeled by nick translation with DIG-dUTP (for 246B23) and biotin-dUTP (for 200E18). Detection was achieved by the use of both antidigoxigenin-rhodamine and avidin-fluorescein antibodies (Vector Laboratories, Peterborough, UK). Chromosomes were counterstained with DAPI (4′,6-diamidino-2-2-phenylindole; Vector Laboratories).

### Histology and immunohistochemistry

Tissues and embryos (E10.5, E12.5 and E14.5) were fixed in 10% neutral buffered formalin for 24 hr, embedded in paraffin and 5 µm sections cut and stained with hematoxylin and eosin. Immunohistochemistry was performed on formalin-fixed, paraffin-embedded embryos using a Cleaved Caspase-3 (Asp175) (Cell Signaling, Hitchin, UK) primary antibody, which was detected using the rabbit VECTASTAIN® Elite ABC Kit (Vector Laboratories, UK) and DAB Substrate Kit for Peroxidase (Vector Laboratories, UK).

### Phenotypic Assays

Complete details of all phenotypic tests performed are listed on the Wellcome Trust Sanger Institute Mouse Resources Portal (http://www.sanger.ac.uk/mouseportal/).

#### Diet challenge

At 4 weeks of age mice were randomly divided into two groups, and either maintained on standard laboratory chow (a ‘normal-fat diet’ containing not less than 9% crude fat, 20% crude protein and 63% carbohydrate; Autoclavable Mouse Breeder Diet 5021, Lab Diet, London, UK) or changed to a ‘high-fat diet’ containing 21.4% crude fat, 17.5% crude protein and 50% carbohydrate (Western RD; Special Diets Services, Essex, UK) until 25 weeks of age.

#### Dual energy X-ray absorptiometry (DEXA)

At 8, 14, 25 or 52 weeks of age, the mice were euthanized, weighed and placed on the PIXImus II Densitometer (GE Medical Systems, Buckingshire, United Kingdom). The PIXImus software package automatically analyses the resulting images, excluding the skull, to calculate body fat mass (g), lean mass (g), fat percentage estimate (%), bone area (cm^2^), bone mineral density (BMD) (g/cm^2^) and bone mineral content (BMC) (g). Quality control using a phantom was performed before imaging. Data was statistically analyzed using two-tailed Student's *t*-test.

### Behavioral tests

Male monosomic *Ms1Dja* mice and male wildtype littermate controls were 3- to 7-month old and were group housed from weaning. All mice were prehandled for 1–2 minutes every day for four days prior to testing for habituation.

#### Elevated plus maze

The elevated plus maze was made of Plexiglas and homogeneously illuminated and tests performed under dim light. The apparatus consisted of four arms (30×5 cm) elevated 40 cm above the floor. Two of the arms contained 15 cm-high black walls (enclosed arms) and the other two none (open arms). Each mouse was placed in the middle section facing an open arm and left to explore the maze for a single 5-minute session with the experimenter out of view. Testing apparatus and areas were cleaned carefully between animals with a solution of 70% ethanol (5%, v/v). All test sessions were recorded and subsequently analyzed with Noldus Ethovision 3 video tracking software (Tracksys Ltd., UK). The tracking system recorded time spent in each arm and number of entries into each arm, considering an animal inside a zone whenever its centre point is within.

#### Social recognition test

The test was performed under red light, using 3 different 2-month old male stimulus mice (C57BL/6NTac/USA; mouse A, 129P2/OlaHsd; mouse B, and C57BL/6NTac/USA; mouse C) sedated with ketamine/xylazine (i.p. 1 g/0.1 g per kg of body weight). Test mice were first habituated for 10 minutes to a test arena identical to their home cage. On day 1, for the habituation-dishabituation test, a stimulus mouse A was placed into the test arena for 1 minute. The same stimulus mouse A was subsequently presented four times at 10 minutes intervals. In the fifth trial, a stimulus mouse B was presented for 1 min. On day 2, for the discrimination test, the animals were simultaneously presented with stimulus mouse A (familiar animal that was encountered on trials 1–4 on day 1) and mouse C (new unfamiliar animal) for 2 minutes. All trials were recorded with an overhead camera and the videos were subsequently scored blind of genotype using a handheld stopwatch. The amount of time the test animal spent investigating, by direct oronasal contact or close approach (∼1 cm), sniffing towards the stimulus mice A and C, was recorded. If the test animal spent longer investigating a novel stimulus animal (mouse C) than the familiar one (mouse A), this was taken as evidence for social recognition. The familiarity ratio was taken as the amount of time [sec] spent investigating the familiar stimulus animal (mouse A) divided by the sum of time spent investigating both stimulus mice A and C. These data are presented as mean ± standard error of the mean and was analyzed using GraphPad Prism software (GraphPad Software Inc.). Statistical analysis for the habituation-dishabituation test data was performed using a two-way ANOVA with repeated measures for trial and genotype as factor and Tukey-HSD *post hoc* test. Discrimination data was analyzed using two-tailed Student's *t*-test using the discrimination ratio.

#### Olfactory function test

Male mice were habituated for 10 minutes to a test arena identical to their home cage and tested in 2×3 minutes trials with a 10 minutes interval. In trial 1, 2×2 cm^2^ filter papers were randomly placed at opposite ends of the arena. One, the control, was swabbed in a clean cage and the other, the stimulus, was swabbed in a cage housing C57BL/6NTac/USA females. The amount of time investigating each swab in 3 minutes was recorded. Male mice with the capacity to perceive odors or pheromones can detect, and prefer to investigate, female-derived olfactory stimuli. In trial two, both filter papers were swabbed in a cage housing C57BL/6NTac/USA female mice. The control swab had 20 µl water added while the stimulus swab had 20 µl of a 1∶100 dilution of almond extract (Dr Oetker, Leeds, UK) in water. Mice with the capacity to smell almond prefer to investigate the swab with the novel supplemental odor. For each trial, the discrimination ratio was calculated as the amount of time (seconds) spent investigating the stimulus swab divided by the sum of time spent investigating both swabs. The data was statistically analyzed using two-tailed Student's *t*-tests.

### RNA extraction and quantitative RT-PCR

Total RNA was extracted from abdominal subcutaneous adipose tissue of 16-week old high fat diet-fed monosomic and control mice (n = 8 per genotype) using TRIzol (Invitrogen, Paisley, UK) according to the manufacturer's instructions. RNA was DNase treated using Turbo DNase (Ambion, Austin, TX) and 1 µg of total RNA was reverse transcribed using the Sprint™ RT Complete kit (Clontech, Mountain View, CA) according to the manufacturer's instructions. Quantitative PCRs performed with SYBR® Green PCR Master Mix (Applied Biosystems) on the ABI 7900HT sequence detection system in accordance with the manufacturer's instructions. Primers are listed in **Table S2**. The qPCR reactions were designed as: 50°C for 2 minutes, 95°C for 10 minutes, 40 cycles of (95°C for 15 seconds and 60°C for 1 minute), followed by 95°C for 15 seconds. The final quantification was determined relative to the average CT of the house-keeping genes *Eif1a*, *Hprt* and *Gapdh* [43]. Statistical analysis was performed using two-tailed Student's *t*-test.

## Supporting Information

Figure S1 **Olfactory function test.** Both monosomic (*Ms1Dja/+*, n = 12) and control (+/+, n = 2) preferentially investigated novel odors when given a choice between an odorized stimulus (left, female odors (FO); right, FO+almond extract) and a control (left, clean cage; right, FO) in sequential trials. In both trials, monosomic (*Ms1Dja/+*) and wildtype (*+/+*) animals were able to distinguish the novel stimulus odor (discrimination ratio >0.5, *P*<0.05 two-tailed Student's *t*-test, the error bars represent the standard deviation of the measurements). There was no significant difference between the discrimination ratios with respect to genotype (*P*>0.05 two-tailed Student's *t*-test, the error bars represent the standard error of the mean), suggesting monosomic (*Ms1Dja/+*) mice are not deficient in detecting either socially relevant (FO) or non-relevant (almond) odors.
(TIFF)

Figure S2 **Quantitative RT-PCR (qRT-PCR) analysis of the genes from the deleted** ***Lipi−Usp25*** **interval in adipocytes from HFD control (*+/+*) and monosomic (*Ms1Dja/+*) mice (n = 8 per genotype at 16 weeks).** Asterisks indicate statistical significance; * *P*<0.05, ** *P*<0.01 (two-tailed Student's *t*-test). The error bars represent the mean with 95% confidence interval.
(TIFF)

Table S1 **A summary of the panel of tests performed on all mice in the study.**
(DOCX)

Table S2 **qPCR primer sequences.**
(DOCX)
