# Correction: Modeling Partial Monosomy for Human Chromosome 21q11.2-q21.1 Reveals Haploinsufficient Genes Influencing Behavior and Fat Deposition

**DOI:** 10.1371/annotation/26a586df-e769-4efc-884f-6e7d448003bc

**Published:** 2012-03-07

**Authors:** Anna M. Migdalska, Louise van der Weyden, Ozama Ismail, Jacqueline K. White, Sanger Mouse Genetics Project, Gabriela Sánchez-Andrade, Darren W. Logan, Mark J. Arends, David J. Adams

The Sanger Mouse Genetics Project was incorrectly abbreviated in the citation. The correct citation is: Migdalska AM, van der Weyden L, Ismail O, White JK, Sanger Mouse Genetics Project, et al. (2012) Modeling Partial Monosomy for Human Chromosome 21q11.2-q21.1 Reveals Haploinsufficient Genes Influencing Behavior and Fat Deposition. PLoS ONE 7(1): e29681. 

