# Supplementary figures and images for: Modeling Partial Monosomy for Human Chromosome 21q11.2-q21.1 Reveals Haploinsufficient Genes Influencing Behavior and Fat Deposition
Source: PLoS One. 2012 Jan 20;7(1):e29681. doi: 10.1371/journal.pone.0029681 (PMC3262805; doi:10.1371/journal.pone.0029681)

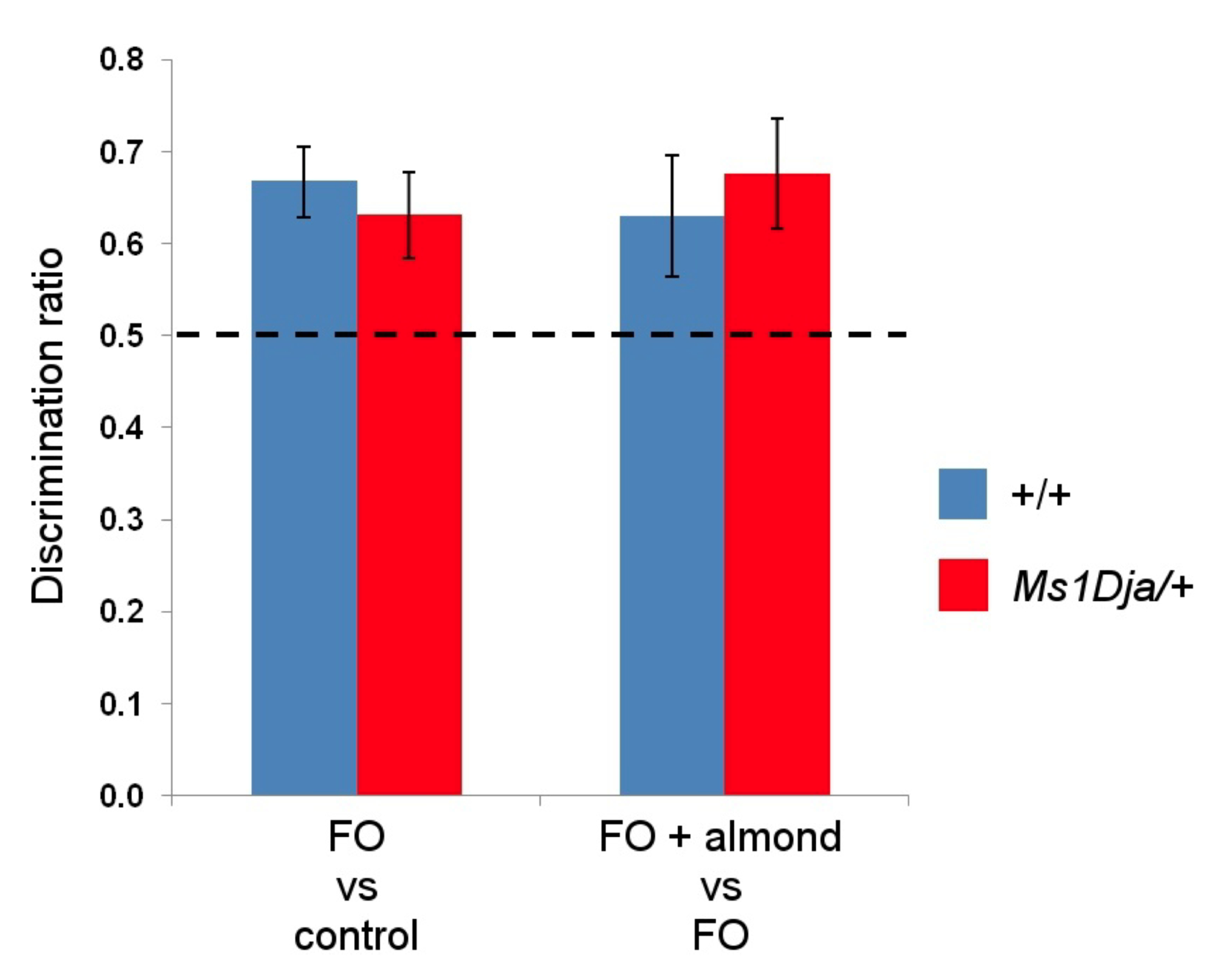

Supplement: Figure S1 — Olfactory function test. Both monosomic (Ms1Dja/+, n = 12) and control (+/+, n = 2) preferentially investigated novel odors when given a choice between an odorized stimulus (left, female odors (FO); right, FO+almond extract) and a control (left, clean cage; right, FO) in sequential trials. In both trials, monosomic (Ms1Dja/+) and wildtype (+/+) animals were able to distinguish the novel stimulus odor (discrimination ratio >0.5, P<0.05 two-tailed Student's t-test, the error bars represent the standard deviation of the measurements). There was no significant difference between the discrimination ratios with respect to genotype (P>0.05 two-tailed Student's t-test, the error bars represent the standard error of the mean), suggesting monosomic (Ms1Dja/+) mice are not deficient in detecting either socially relevant (FO) or non-relevant (almond) odors. (TIFF) [file pone.0029681.s001.tif]

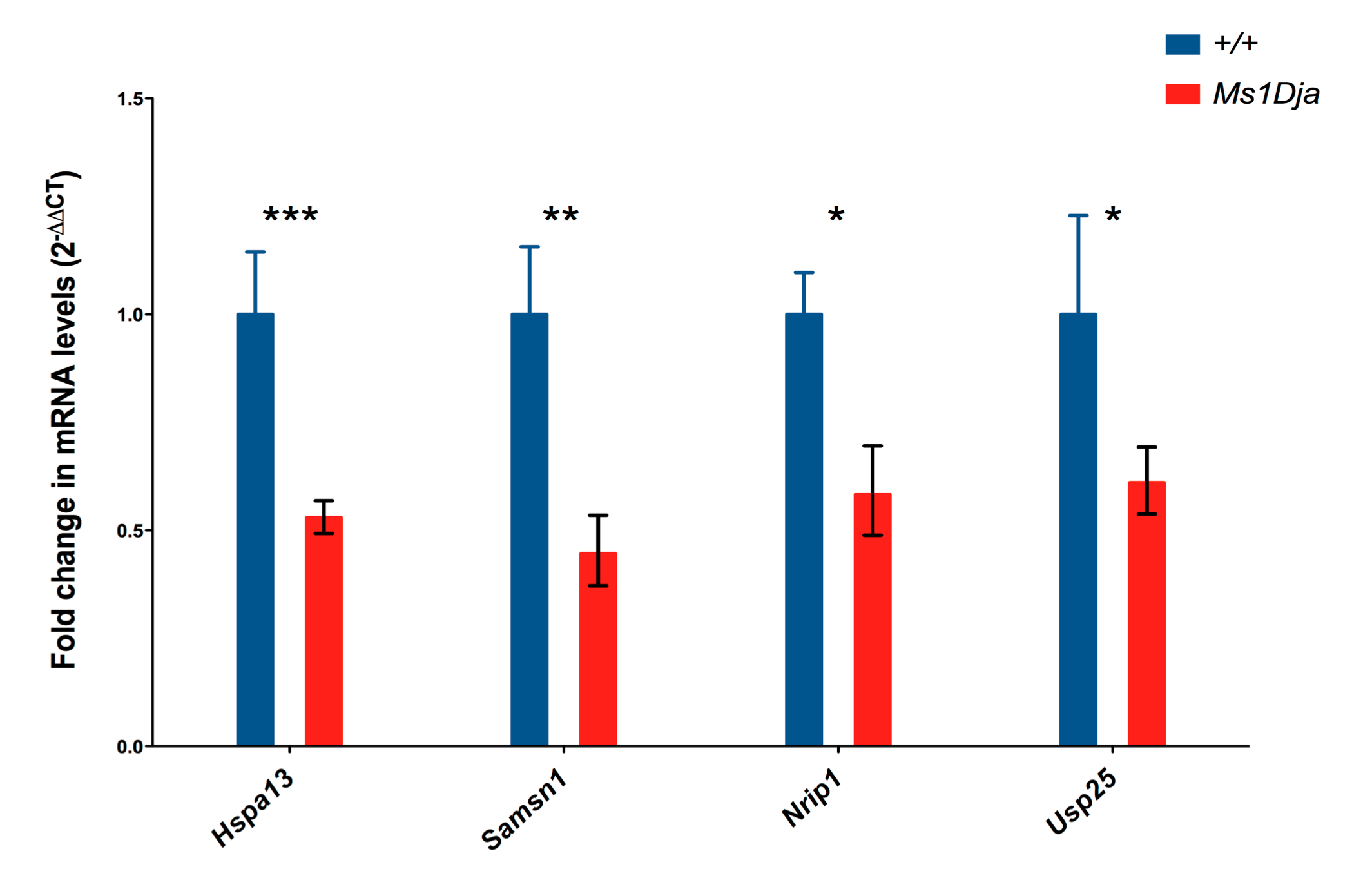

Supplement: Figure S2 — Quantitative RT-PCR (qRT-PCR) analysis of the genes from the deleted Lipi−Usp25 interval in adipocytes from HFD control (+/+) and monosomic (Ms1Dja/+) mice (n = 8 per genotype at 16 weeks). Asterisks indicate statistical significance; * P<0.05, ** P<0.01 (two-tailed Student's t-test). The error bars represent the mean with 95% confidence interval. (TIFF) [file pone.0029681.s002.tif]
