# Supplementary material for: Modeling Partial Monosomy for Human Chromosome 21q11.2-q21.1 Reveals Haploinsufficient Genes Influencing Behavior and Fat Deposition
Source: PLoS One. 2012 Jan 20;7(1):e29681. doi: 10.1371/journal.pone.0029681 (PMC3262805; doi:10.1371/journal.pone.0029681)
Supplement: Table S1 — A summary of the panel of tests performed on all mice in the study. (DOCX) [file pone.0029681.s003.docx]

**Supplementary Table 1: A summary of the panel of tests performed on all mice in the study.**

| **Age of mouse** | **Test name** | **Assessment** |
| --- | --- | --- |
| 4-week old | Weighing | Monitor body weight (done on a weekly basis) |
| 4-week old | Hair dysmorphology | Check coat formation and development |
| 6-week old | Hair follicle cycling | Investigate cycling of the hair follicles |
| 9-week old | Open field | Measure locomotion, habituation and fear/anxiety responses to a novel environment |
| 9-week old | Modified SHIRPA | Assess the presence of any gross motor and neurological abnormalities |
| 9-week old | Grip strength | Assess neuromuscular function and muscular strength of the paws |
| 10-week old | Dysmorphology | Assess the presence of any gross morphological abnormalities |
| 10-week old | Hot plate | Assess thermal pain perception |
| 12-week old | Indirect calorimetry | Investigate metabolism, circadian pattern and behavior |
| 13-week old | Glucose tolerance | Obtain fasted basal blood glucose concentrations & investigate glucose tolerance and clearance |
| 14-week old | Auditory brainstem response | Assess hearing sensitivity across a broad range of frequencies |
| 14-week old | Dual energy X-ray absorptiometry | Obtain body composition and bone mineral density data |
| 14-week old | X-ray imaging | Obtain the high-resolution X-ray images of the skeleton |
| 15-week old | Core temperature stress | Obtain basal body temperature and assess stress-induced hyperthermia |
| 15-week old | Eye morphology screen | Assess the presence of gross morphological abnormalities in the eye |
| 16-week old | Heart weights | Assess the weight of the heart |
| 16-week old | Hematology & plasma chemistry | Assess variable whole blood parameters and plasma clinical chemistry parameters |
