# Supplementary material for: Modeling Partial Monosomy for Human Chromosome 21q11.2-q21.1 Reveals Haploinsufficient Genes Influencing Behavior and Fat Deposition
Source: PLoS One. 2012 Jan 20;7(1):e29681. doi: 10.1371/journal.pone.0029681 (PMC3262805; doi:10.1371/journal.pone.0029681)
Supplement: Table S2 — qPCR primer sequences. (DOCX) [file pone.0029681.s004.docx]

**Supplementary Table 2: qPCR primer sequences.**

| **Gene** | **Forward: 5’ to 3’** | **Reverse: 5’ to 3’** |
| --- | --- | --- |
| *Lipi* | GCTTGACATACTTTCAGTCCACA | GCGTGTTACATTCATCTGGTTGA |
| *Rbm11* | GACCGTGTTTGTGGGCAATTT | CTTCGGCTTTCCGTCTCTGT |
| *Hspa13* | AGGTTGACGTGTTCTACGTGT | CCAGACATCGCTCGTGTTAGG |
| *Samsn1* | CCAAGTCCCTATGACACCGAC | CCTGGATAGTCTGGTGGTTCT |
| *Nrip1* | AGACCAGAACTTTAACCTCTCGG | CGATGGAATCAGACAGCCTCT |
| *Usp25* | CAGAAGCACCAGCAGACATTT | TGGCATTCTTTGCAGTGAGGA |
| *Gapdh* | AGGTCGGTGTGAACGGATTTG | TGTAGACCATGTAGTTGAGGTCA |
| *Hprt1* | TCAGTCAACGGGGGACATAAA | GGGGCTGTACTGCTTAACCAG |
| *Eif1a* | AAAAACAGGCGCAGAGGTAAA | TCCTCACACCGTCAAAGCAC |
